# Risk factors for in-brain local progression in elderly patients after resection of cerebral metastases

**DOI:** 10.1038/s41598-019-43942-9

**Published:** 2019-05-15

**Authors:** Christopher Munoz-Bendix, Marion Rapp, Hendrik-Jan Mijderwijk, Christiane von Sass, Maxine Dibué-Adjei, Jan Frederick Cornelius, Hans-Jakob Steiger, Bernd Turowski, Michael Sabel, Marcel A. Kamp

**Affiliations:** 1 Department of Neurosurgery, Heinrich Heine University Hospital, Dusseldorf, Germany; 2 Institute for Diagnostic and Interventional Radiology, Heinrich Heine University Hospital, Dusseldorf, Germany

**Keywords:** Surgical oncology, CNS cancer, CNS cancer, Metastasis, Cancer therapy

## Abstract

Intracranial metastases are the most frequent brain tumor with recurrence rates after treatment of around 40–60%. Age is still considered a determinant of treatment and prognosis in this pathology. Recent studies analyzing the impact of metastasectomy in elderly patients focused on reporting perioperative mortality and morbidity rates but not on the evaluation of oncological outcome parameters. Aim of this study is to determine risk factors for in-brain local recurrence after brain surgery in this sub-population. From October 2009 until September 2016 all patients aged 65 years and above with histopathologically confirmed metastasis after surgical resection were retrospectively studied. Clinical, radiological and perioperative information was collected and statistically analysed. Follow-up consisted of clinical and radiological assessment every 3-months following surgery. 78 patients were included, of these 50% were female (39 patients). Median age was 71 years (66–83). Early postoperative-MRI verified a complete surgical resection in 41 patients (52.6%) and showed a tumor-remnant in 15 patients (19.2%). In 22 patients the MRI result was inconclusive (28.2%). None of the patients experienced severe complications due to surgery. The median postoperative NIHSS was adequate 1 ± 1.4 (0–6), nonetheless, insignificantly improved in comparison to the preoperative NIHSS (p = 0.16). A total of 20 patients (25.6%) presented local recurrence. The only statistically significant factor for development of local in-brain recurrence after resection of cerebral metastases in patients above 65 years of age was a tumor-remnant in the early postoperative MRI (p = 0.00005). Median overall survival was 13 months. Local in-brain recurrence after surgical resection of a cerebral metastasis in patients above 65 years of age was 25.6%. In our analysis, tumor-remnant in early postoperative MRI is the only risk factor for local in-brain recurrence. Oncological parameters in the present cohort do not seem to differ from recent phase III studies with non-geriatric patients. Nevertheless, controlled studies on the impact of metastasectomy in elderly patients delivering high quality reliable data are required.

## Introduction

Although the exact incidence of cerebral metastases from solid cancers is unknown, intracerebral metastases are the most frequent brain tumors with a 3–5 times higher incidence than newly diagnosed primary malignant brain tumors each year^1,2^. Incidence of cerebral metastases was considered to increase from 2.8–11.1 per 100,000 population in the years before 1990 to an incidence of 7–14.3 per 100,000 population in more recent studies^1^. Cumulative incidence of cerebral metastases may be age-related as the highest cumulative incidence is observed in patients with primary breast cancer at the age between 20 and 39 years, in lung cancer patients at the fifth decade and in malignant melanoma patients at the sixth decade of life^3^. Cumulative incidence is considered to be lowest for all primary cancers in the age group above 70 years, with exception of melanoma^3^.

Despite the presumably lower incidence of cerebral metastases in elderly patients, incidence in this subgroup increases due to the high number of elderly patients, general increase of occurrence of cerebral metastases, improved diagnosis of brain metastases and better treatment of the primary cancer. Moreover, age above 60 years was one major risk factor for impaired overall survival (OS) in an early prospective randomized study comparing combined treatment of surgery and adjuvant whole-brain radiation therapy (WBRT) with an exclusive WBRT^4^. A recent individual patient data meta-analysis of 3 randomized trials of stereotactic radiosurgery (SRS) with or without WBRT for 1 to 4 cerebral metastases suggested that age might be a factor influencing the efficiency of an adjuvant WBRT following SRS. For patients <50 years of age, SRS alone favoured survival and an additional WBRT did not impact the distant in-brain progression rate. Adjuvant WBRT significantly decreased the risk of new cerebral metastases without affecting the OS in patients aged >50 years^5^. Some recent retrospective studies reported age as a risk factor for a reduced survival^6^. Age is therefore still considered to be a determinant of treatment and prognosis in this pathology in recent guidelines^7^.

The Dutch prospective and randomized study (surgery and WBRT vs. WBRT alone) identified age as a major determinant for OS^4,8^. However, patients included in this study were recruited between 1985 and 1991, a preoperative MRI to diagnose single intracerebral metastases was not mandatory and histological confirmation of the presumed metastasis was not necessarily required prior to treatment. Since the end of the 1980s, advancements in pre- and postoperative diagnosis and surgical techniques have been made^9–19^. Recent studies analyzing the impact of metastasectomy in elderly patients focused on reporting perioperative mortality and morbidity rates but not on evaluation of oncological outcome parameters.

Aim of the present retrospective study was therefore to analyze the progression-free and overall survival, rate of local in-brain progression and complications after surgery of brain metastases in elderly patients over the age of 65.

## Methods and Materials

### Study design, inclusion and exclusion criteria

This study represents a clinical and radiological retrospective analysis of a consecutive series of patients treated for intracranial metastases at a large European tertiary care centre. This study involved the review of clinical records as part of medical care. We retrospectively studied and analysed medical records and their corresponding radiological diagnostic tests of every patient presenting with histologically confirmed brain metastases from October 2009 until September 2016.

Inclusion criteria were: (1) histological diagnosis of an intracranial secondary tumor, (2) operated only at our institution, (3) between October 2009 and September 2016, (4) pre- and postoperative MRI (pre- and post gadolinium-enhanced T1-weighted sequences, T2-weighted sequences and Fluid Attenuated Inverse Recovery (FLAIR)), (5) clinical and radiological follow-up at our institution, (6) age older than 65 years.

Exclusion criteria included: (1) other tumor than cerebral metastases (primary brain tumor, small cell lung cancer, neuroendocrine or sarcoma metastases and lymphoma) (2) prior surgical treatment at a different institution, (3) exclusively palliative or no neuro-oncological treatment, (4) previous treatment with the following: biopsy, stereotactic biopsy, radiotherapy and/or SRS, and (5) preoperative diagnosis of leptomeningeal carcinomatosis (LC).

### Surgery

All patients included in this study received surgical treatment for one cerebral metastases, although patients with more than one metastasis were also considered for surgery. Indication for surgical treatment of one cerebral metastasis in patients with 2 or more cerebral metastases was (1) symptomatic lesions, (2) mass effects (3) no history of systemic disease or unclear diagnosis, (4) intratumoral hemorrhage, and (5) large posterior fossa tumors.

Intraoperative frozen sections were obtained in all patients. After the histological diagnosis of a cerebral metastasis by frozen section, standard white-light assisted – and if possible – *en bloc* circumferential resection was performed. Surgery integrated the intraoperative use of ultrasound (ProSound alpha7, Hitachi Aloka Medical America Inc., U.S.A.), neuro-navigation (Brainlab Navigation System, Brainlab AG, München, Germany) and awake surgery using an asleep-awake-asleep protocol as described before for patients with eloquent located cerebral metastases^11^. An eloquent brain region was defined as a cortical or subcortical brain area where we expect intraoperative stimulation to elicit changes in neurologic conditions (particularly regarding speech, movement and tactile sensation) or to elicit a response in electrophysiological recordings in corresponding areas^11,20^. Adjuvant therapy was individually decided upon in every case after histological diagnosis in an interdisciplinary tumour board. Recommendations for adjuvant radiation depended on various parameters such as number of cerebral lesions, degree of resection, Karnofsky Performance Scale (KPS) and the patient preference.

### Data collection, follow-up and definition of outcome measures

Additionally, pre- and postoperative clinical characteristics of the patients, preoperative performance scale, localization, number of metastases, characteristics and classification of the tumor, treatment and incidence of each primary tumor, extent of surgical resection, fluorescence of the tumor, use of intraoperative monitoring, perioperative complications, periodically follow-up visits, recurrence, time to recurrence, loco-regional or distant metastases, neoadjuvant/adjuvant/palliative therapy, survival, cause and date of death (if applicable) were collected from charts and electronic records and analyzed.

Pre-, postoperative and follow up clinical assessments were standardized using the National Health Institute of Stroke Scale (NIHSS). Degree of surgical resection was verified by early postoperative 1.5T-MRI as described before^16^. A senior neurosurgeon and neurological radiologist performed the radiological analysis. After surgery, patients were followed-up including a contrast-enhanced MRI every 3 months.

Local in-brain-progression/recurrence was defined according to the RANO criteria^21^, as an increase by 20% from the initial longest diameter of the target lesion with an absolute 5 mm growth, as measured in contrast-enhanced T1, T2 and diffusion sequences. Radiological postoperative evaluation of the resection cavity was defined as inconclusive when characteristics such as postoperative blood residues, pronounced vessels, reactive tissue, suboptimal image quality were present^16^. Distant in-brain-progression was defined as appearance of new metastasis distant to the site of the resected metastasis (distance to the resection cavity of at least 2 cm). Dural inclusion of cerebral metastasis and leptomeningeal carcinomatosis (LC) were interpreted as different radiological entities. Dural inclusion explicitly represented radiological or intraoperatively verified contact of the dura with the brain metastases (BM) with no additional radiological signs of LC. LC was diagnosed by cranial MRI showing a diffuse enhancement of meninges or by lumbar puncture and confirmation of malignant tumour cells in the cerebrospinal fluid (CSF). Time to in-brain-progression was defined as time between surgery and diagnosis of the in-brain-tumour progression. The overall survival was considered as time span between surgery and death.

### Statistical analysis

Follow-up ended in April 2018 and the database was finalized shortly thereafter. All statistical analyses were performed with SPSS software (Version 22.0, -IBM-, USA). Data is presented as the median and standard deviation. Descriptive statistics including mean and standard error of mean were calculated for all continuous variables. The Chi-Square-test was used in nominal variables to identify significant differences. Contingency tables were performed according to the number of possible answers. As multiple statistical testing was performed, the significance level according to Šidák’s and Bonferroni’s correction: Šidák’s and Bonferroni-correction revealed an adjusted p-value of 0.0051 or 0.005, respectively.

### Ethical statement

Data acquisition, radiological interpretation, as well as an analysis of both, were approved by the institutional research ethics board (Medical Faculty, Heinrich-Heine- University, Nr. 5713). For every patient treated at our institution with any brain pathology we obtained an informed consent allowing a retrospective analysis of the data, as well as the inclusion of the pathological specimen in an institutional tumor-bank.

## Results

### Patient’s characteristics

A total of 78 patients aged 65 or above were included; of these 50% were female (39 patients). The median age was 71 years (66–83). In our series, 50 patients (64.1%) presented a single metastasis, 18 patients (23.1%) had two or three cerebral metastases and 10 patients (12.8%) presented more than three metastases. All patients were required to be in good clinical condition to be eligible for surgery. Every patient had a preoperative Karnofsky Performance Scale (KPS) of 70 or more. The median preoperative KPS was 90 ± 9.8 and the median pre-operative NIHSS was 1 ± 1.9 (0–10).

The most common primary tumor type was non-small cell lung carcinoma (NSCLC) in 35 patients (44.9%). Adenocarcinoma was the most frequent histological diagnosis and was present in 58 patients (74.4%). Epidemiological features of the present patient population are summarized in Table 1.

**Table 1:** Epidemiological data.

	No. patients	%	Local In-Brain Recurrence p-value	Distant In-Brain Recurrence p-value
**Gender**				
Male	39	50		
Female	39	50		
**Age**				
median age (years)	71			
range (years)	66–83			
**Number of Metastases**			0.8665*	N/A
1	50	64.1		
2/3	18	23.1		
>3	10	12.8		
**Primary site**			0.4228*	0.7897*
NSCLC	35	44.8		
Malignant melanoma	9	11.5		
Breast Cancer	8	10.3		
Renal Cancer	6	7.7		
Gastrointestinal Cancer	10	12.8		
Urogenital Cancer	4	5.1		
Other	6	7.7		
**Histology**			0.4853*	0.2139*
Adenocarcinoma	58	74.4		
Malignant melanoma	9	11.5		
Clear cell carcinoma	4	5,1		
Others	7	9.0		
**Localization**				
Supratentorial	59	75.6		
Infratentorial	11	14.1		
Both	8	10.3		
**Surgical technique**			0.5675*	0.7929*
En bloc resection	40	51.3		
Peace-meal resection	38	48.7		
**Use of intraoperative neurophysiological monitoring**				
yes	40	51.3		
no	38	48.7		
**Degree of surgical resection on MRI**		0.00005*	0.3471*	
complete	41	52.5		
incomplete	15	19.2		
questionable	22	28.2		
**Adjuvant radiation therapy**				
Whole-brain radiation therapy	37	47.4		
stereotactic radiosurgery	14	18		
local fractionated radiation	10	12.8		
WBRT & SRS	2	2.6		
no radiation	15	19.2		
	78			
**Local in-brain progression**				
yes	20	25.6		
no	58	74.4		
**Distant in-brain progression**				
yes	21	26.9		
no	57	73.1		
**Leptomeningeal carcinosis**				
yes	13	16.7		
no	65	83.3		

*Chi Square Test.

### Treatment

Surgery was performed as an en-bloc resection in 40 patients (51.2%) and as piece-meal resection in 38 patients (48.7%). Early postoperative-MRI verified a complete removal in 41 patients (52.6%) and showed a tumor-remnant in 15 patients (19.2%). In 22 patients the MRI result was inconclusive (28.2%).

**Figure 1:**
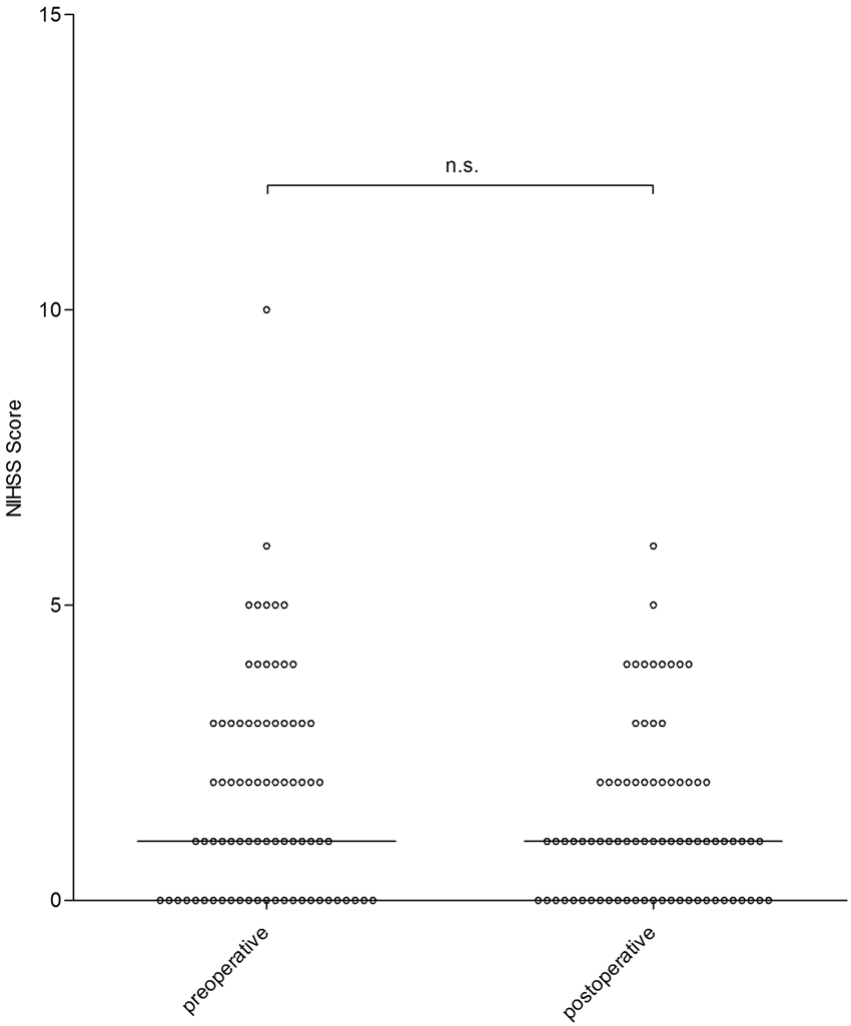
Pre- and postoperative NIHSS. shows the pre- and postoperative NIHS Scores which were not significantly different.

No patient experienced a severe complication due to surgery, such as surgery-associated death or cardio-pulmonary complications. The median post-operative NIHSS was 1 ± 1.4 (0–6). The median post-operative NIHSS was 1 ± 1.4 (0–6). Therefore, median post-operative NIHSS was not significantly improved in comparison to the pre-operative NIHSS (p = 0.16; Fig. 1) The NIHSS improved after surgery in 28 patients (35.9%), decreased in 9 patients (11.6%) and was unchanged in 41 patients (52.6%).

As local adjuvant treatment, almost half of the patients received whole brain radiation therapy (37 patients, 47.4%) and in 15 patients no adjuvant therapy was performed (summarized in Table 1).

### Local in-brain progression and overall survival

A total of 20 patients above 65 years of age (25.6%) presented a local recurrence with a median time-to-local recurrence of 3 ± 2.9 months (0–10 months). 23 patients (26.9%) developed distant metastases and 13 patients (16.7%) carcinomatous meningitis. (See Table 2).

**Table 2:** Recurrence rates.

	No. of Patients	Mean (SD)*	min/max*
**Overall Survival-Follow-Up - No. (%)**			
	26 (30.2)	12 (14)	0/74
**Progression Free Survival - No. (%)**			
	26 (30.2)	9 (14)	0/74
**Local Recurrence - No. (%)**			
*No*	65 (30.5)		
*Yes*	21 (9.8)		
**Time-to-Local Recurrence - No. (%)**			
	21 (9.8)	6 (5)	0/22
**Distant Recurrence - No. (%)**			
*No*	63 (29.6)		
*Yes*	23 (10.8)		
**Time-to-Distant Recurrence - No. (%)**			
	23 (10.8)	7 (7)	0/35
**Carcinomatous Meningitis - No. (%)**			
*No*	72 (33.8)		
*Yes*	14 (6.6)		
**Time-To-Carcinomatous Meningitis - No. (%)**			
	14 (6.6)	8 (10)	0/46

*Months.

According to statistical correlation, the only factor statistically significant for the development of local in-brain recurrence after resection of cerebral metastases in patients above 65 years of age was a tumor-remnant in the early postoperative MRI (p = 0.00005). In our series no other risk factors such as sex, localization, number of metastases, preoperative KPS and NIHSS, postoperative NIHSS, primary tumor site, histology, type of surgical resection and type of adjuvant radiation, could be identified (each p > 0.05).

**Figure 2:**
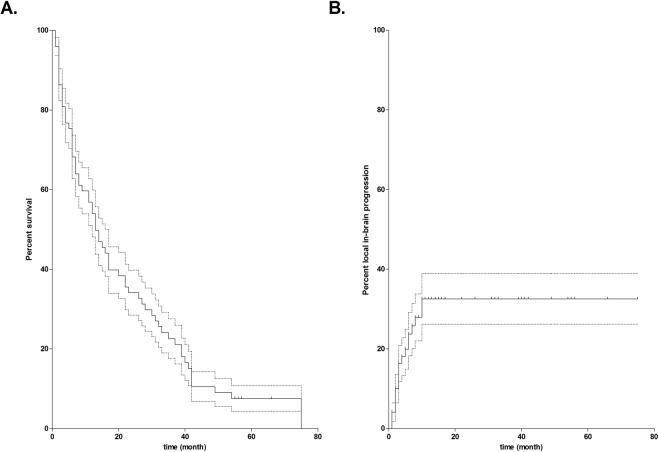
Progression-free and overall survival. shows the Kaplan Meier estimates for overall survival (**A**) and progression free (**B**) with its 95%-confidence intervals (dotted lines) over time in patients above 65 years after surgical resection of cerebral metastases.

A total of 8 patients (10.3% on May 22^nd^ 2018) were still alive at the end of the study and continued with the scheduled follow-up visits. Two of these 8 patients suffered from local recurrence. The median OS was 13 months. Kaplan Meier estimates for OS and local in-brain progression are shown in Fig. 2.

## Discussion

The main results of our analysis are as follows: (1) the local in-brain recurrence after surgical resection of a BM in patients above 65 years of age is 25.6% with a median time to occurrence of three months; (2) in patients above 65 years of age tumor-remnant in an early postoperative MRI was the only risk factor for local in-brain recurrence and (3) the median overall survival was 13 months in the present series.

Most studies analyzing the impact of cerebral metastasis resection focus on reporting perioperative morbidity and mortality rates but omit oncological outcome parameters such as (local) in-brain progression and survival. Median overall survival was 13 months after metastasectomy in the present retrospective series of elderly patients aged 65 years and over. It ranged between 2.8 and 18 months in recent prospective randomized and controlled phase III trials including surgery as treatment of cerebral metastases (e.g. 11.6 and 12.2 months in the NCCTG N107C/CEC·3 trial by Brown *et al*., 2017; 17 and 18 months in the study by Mahajan *et al*. 2017; 2.8 months in the trial by Roos *et al*., 2011, 10.7 and 10.9 months in the EORTC 22952–26001 study by Kocher and coworkers, 2011, respectively). Although results from retrospective studies have limited comparability to those derived from prospective randomized and controlled phase III trials, median survival in the present analysis seems to be comparable to those in recent phase III studies. However, overall survival was related to the treatment of cerebral metastases only in early phase III trials from the 1990s (Patchell *et al*., 1990; Vecht *et al*., 1993) but not in more recent phase III trials (e.g. Kocher *et al*., 2011; Brown *et al*., 2017; Mahajan *et al*., 2017). Occurrence of single cerebral metastasis and the choice of their treatment modalities may therefore be insufficient to predict survival of patients – even in elderly patients. In contrast, treatment of cerebral metastases is well known to influence the local and distant in-brain progression as well as patients’ quality of life. In the present study, local recurrence rate was 25.6%, the distant development rate was 26.9%. These rates are congruent with our previous results and the recurrence rates reported in prospective randomized and controlled phase III trials. Therefore, elderly patients have comparable in-brain progression and overall survival as reported from previous oncologic patient cohorts. After thorough analysis, tumor-remnant in the early postoperative MRI as described in^16^ was the only statistically significant risk factor for local recurrence. Relevance of early postoperative MRI in oncological patients has already been defined^16,22^. Although many factors (e.g. surgical technique, number of metastases, local control) have been proposed as the cause of local recurrence and distant development or carcinomatous meningitis^22–27^, we could not establish another association or correlation in patients above 65 years of age.

In the present population, we observed no severe complication and no case fatalities within the first 30 days after surgery. The pre- and postoperative NIHSS, as well as KPS and follow-up visits showed no immediate or mediate deteriorations or complications. The median pre- and postoperative NIHSS was 1 without significant differences suggesting no new neurological deficits due to metastasectomy and a favorable overall surgical outcome. Perioperative morbidity and mortality were considered to be elevated in elderly patients in some neurosurgical but non-oncological series [e.g.^28^]. However, this may only partially be true for geriatric patients with cerebral metastases. Within a retrospective analysis of a United States inpatient sample, 4.907 patients aged 64 years and above were identified who underwent brain metastases resection. This study concluded that surgical resection of brain metastases among the elderly up to the ninth decade of life is feasible but that age above 80 years and comorbidities were important prognostic factors for inpatient outcome^29^. In a retrospective observational cohort-comparison study of patients with brain metastases, complication rate was 5.7% in the geriatric cohort with 174 patients aged 70 years and over^30^. Several further studies reported low complication rates after surgery of elderly patients with malignant brain tumors^31–34^.

### Limitations

Our study presents a single center experience with a reasonable number of patients, with a homogenous diagnostic and therapeutic approach allowing comparison. However, our study presents some limitations: (1) 78 geriatric patients suffering from cerebral metastases within a period of 7 years were included. This is based on a very dense net of exclusion criteria. Therefore, this cohort might not be representative for all geriatric patients. However, the present cohort is heterogeneous in terms of different primary sites and adjuvant therapies. Furthermore, a subgroup analysis for patients with urgent or acute surgery for BM was not performed. Due to the acute setting, proper planning and supplementary accessories might have been impossible to accomplish and thus have resulted in a higher probability of tumor-remnant or an increased risk of complications. (2) Extent of surgical resection was analysed by an early postoperative-MRI. In the current literature, only few retrospective studies analysed the impact of this method in diagnosing residual tumor tissue^16,35^. A definite conclusion regarding the resection degree was not possible in 28.2% for several reasons, e.g. residual tumor tissue could not reliably be differentiated from dilated vessels in the wall of the resection cavity, poor image quality (e.g. due to patient motion), blood in the resection. The early postoperative MRI revealed an incomplete surgical resection is in 19.2%. The comparatively high rate of incompletely and questionably completely resected metastases is in line with previous non-geriatric series^16^. (3) A median time-to-local recurrence of 3 ± 2.9 months (0–10 months) is fairly low. Time-to-local in brain-progression was therefore lower as in the recent phase III trials (e.g. 7.6 months and not reached in the recent phase III trial by Mahajan *et al.*)^36^. The reason for the comparatively low time-to-local in-brain progression remains unclear. One explanation might be the high rate of incomplete surgical resection or patients without any adjuvant radiation therapy (19.2% each) and the significant correlation between verification of tumor remnants on an early MRI and a later local in-brain progression. We are not aware of any studies directly analyzing a potential correlation between local recurrence and death. Several recent phase III studies addressed the local control and/or the overall survival after treatment of 1–4 cerebral metastases. Except the study by El Gantery and coworkers (2014), none of the studies observed an effect of the therapy modality on the overall survival but nearly all studies showed a significant effect on the local control^9,12,29,30,37^. (4) Current prognostic indicators were not performed or analyzed in our population. A comparative analysis between our data and known literature cannot be performed. (5) Moreover, preoperative evaluation of the geriatric population should be required in order to increase quality of care, identify unknown entities, reduce complications and improve outcome^38^. There are many tools to preoperatively assess geriatric patients. Although there are still some discrepancies^39^ and new innovative tools are being studied^40^, the most common and thorough tool utilized to identify those patients with higher risk for worse outcome or a greater benefit from surgical treatment is the comprehensive geriatric assessment (CGA)^41^. Interestingly, most of the tools (if not all) use the KPS described in 1948 as a base^42^. Still, a unified guideline for this subgroup of patients has yet to be established. In our study, we did not perform any geriatric assessment. Nonetheless, the pre- and postoperative NIHSS, as well as KPS and follow-up visits showed no immediate or mediate deteriorations or complications, and more importantly, they represent an adequate parameter. However designed for other reasons and purposes^37,43,44^, the NIHSS seems a feasible tool. (5) The influence of comorbidities, multi-organ metastatic disease, current medication and other elective or palliative surgeries was not taken into account. (7) Controlled or absent systemic neoplastic disease was assumed according to the clinical status and routine blood work. No evidence was established to prove that fact. (8) Another subgroup analysis of patients, in whom a palliative intervention therapy was performed, was also not analyzed separately. How this affects the course of disease, the type of therapy received additionally or the influence on overall survival remains unknown. (9) Patients’ adherence, compliance, complications or changes altogether regarding adjuvant therapy were not analyzed. (10) Preoperative elaborate analysis of geriatric patients was not performed.

## Conclusion

Local in-brain recurrence after surgical resection of a BM in patients above 65 years of age was 25.6%. Tumor-remnant in early postoperative MRI is the only risk factor for local in-brain recurrence. Mean overall survival was 13 months. Oncological parameters in the present cohort seem not to differ from recent phase III studies with non-geriatric patients. As reliable data are lacking, controlled studies analyzing the impact of metastasectomy in elderly patients are required.

### Disclosure of potential conflicts of interest

Prof. Sabel and PD Dr. Rapp work as consultants for Johnson & Johnson Company and Integra Company. Dr. Dibué-Adjei is an employee of LivaNova PLC, manufacturer of vagus nerve stimulators. All other authors certify that they have no affiliations with or involvement in any organization or entity with any financial interest (such as honoraria; educational grants; participation in speakers’ bureaus; membership, employment, consultancies, stock ownership, or other equity interest; and expert testimony or patent-licensing arrangements), or non-financial interest (such as personal or professional relationships, affiliations, knowledge or beliefs) in the subject matter or materials discussed in this manuscript.
